# Comparative Genomics Unveils Functional Diversity, Pangenome Openness, and Underlying Biological Drivers among *Bacillus subtilis* Group

**DOI:** 10.3390/microorganisms12050986

**Published:** 2024-05-14

**Authors:** Taiquan Wang, Yiling Shi, Mengzhuo Zheng, Jinshui Zheng

**Affiliations:** 1 National Key Laboratory of Agricultural Microbiology, Huazhong Agricultural University, Wuhan 430070, China; taiquanwang@webmail.hzau.edu.cn (T.W.); syl1@webmail.hzau.edu.cn (Y.S.); mengzhuo2017@webmail.hzau.edu.cn (M.Z.); 2 Hubei Key Laboratory of Agricultural Bioinformatics, College of Informatics, Huazhong Agricultural University, Wuhan 430070, China

**Keywords:** comparative pangenomics, *Bacillus subtilis* group, pangenome openness, functional enrichment, *Bacillus subtilis*, mobile elements

## Abstract

The *Bacillus subtilis* group (Bs group), with *Bacillus subtilis* as its core species, holds significant research and economic value in various fields, including science, industrial production, food, and pharmaceuticals. However, most studies have been confined to comparative genomics analyses and exploration within individual genomes at the level of species, with few conducted within groups across different species. This study focused on *Bacillus subtilis*, the model of Gram-positive bacteria, and 14 other species with significant research value, employing comparative pangenomics as well as population enrichment analysis to ascertain the functional enrichment and diversity. Through the quantification of pangenome openness, this work revealed the underlying biological drivers and significant correlation between pangenome openness and various factors, including the distribution of toxin–antitoxin- and integrase-related genes, as well as the number of endonucleases, recombinases, repair system-related genes, prophages, integrases, and transfer mobile elements. Furthermore, the functional enrichment results indicated the potential for secondary metabolite, probiotic, and antibiotic exploration in *Bacillus licheniformis*, *Bacillus paralicheniformis*, and *Bacillus spizizenii*, respectively. In general, this work systematically exposed the quantification of pangenome openness, biological drivers, the pivotal role of genomic instability factors, and mobile elements, providing targeted exploration guidance for the Bs group.

## 1. Introduction

*Bacillus* is vast and species-rich and possesses considerable economic and application value [1]. In 2020, its market value reached USD 18 billion, with an anticipated annual growth rate of approximately 8.7% by 2026 [2]. Industrially, *Bacillus* are utilized to produce a variety of compounds and products, including enzymes, peptide antibiotics, surfactin, biofertilizers, chemicals, pharmaceuticals, and nutraceuticals [3,4]. *Bacillus subtilis*, the model of Gram-positive bacteria, holds a significant historical and research status.

Since its first description by Ferdinand Cohn in 1872, the diversity and habitat distribution of *B. subtilis* have been extensively documented [5,6,7]. Generally recognized as safe (GRAS) by the United States due to its stability and non-toxicity to humans, *B. subtilis* is widely used in pharmaceutical and food fermentation [8,9,10]. Recent studies have shown that overexpressing proline transport proteins in *B. subtilis* and co-culturing with *Corynebacterium glutamicum* can significantly enhance the production of fengycin [11]. Additionally, a metabolic pathway in *B. subtilis* for D-tagatose production from D-galactose has been engineered, contributing to its application in food and pharmaceutical industries [12].

The *Bacillus subtilis* group (Bs group) comprises a complex of species that are challenging to differentiate based on phenotypic characteristics alone, and it is not recognized as a standardized taxonomic concept due to fluctuating membership across different taxonomic ranks [13,14,15]. Recently, it was reported that, in addition to *B. subtilis*, the group also comprised other species, including *B. amyloliquefaciens*, *B. atrophaeus*, *B. mojavensis*, *B. paralicheniformis*, *B. tequilensis*, *B. vallismortis*, and others [16]. The application of the Bs group aligns with the theme of green and sustainable development, as well as the active measures regarding the low-carbon economy and sustainability taken by the European Union [17]. Members of the Bs group, such as *B. amyloliquefaciens* and *B. licheniformis*, are extensively involved in the production of enzymes, vitamins, antibiotics, amino acids and their derivatives, pharmaceuticals, or nutraceuticals [18,19] and play a vital role in production. Market reports suggested that the enzyme market size was USD 11.47 billion in 2021, with a projected annual growth rate of 6.5% until 2030 [2]. *Bacillus* is identified as the predominant source of the key enzyme protease, a crucial constituent in enzyme formulations [20]. Furthermore, the Bs group is a significant component of the probiotics market, with some strains of Bs, *B. amyloliquefaciens*, and *B. licheniformis* explicitly reported as probiotics [21,22]. The global probiotics market share was valued at USD 61 billion in 2021, with *Bacillus* probiotics accounting for USD 7.6 billion, representing 12.5% of the global market “https://www.marketsandmarkets.com/Market-Reports/probiotics-market69.html (accessed on 9 May 2024)”.

In summary, members of the Bs group hold a pivotal position in applications and industrial production [23]. However, within the investigation scope of the Bs group, there was a scarcity of research systematically comparing and exploring functions based on different species group scales. Traditional comparative genomics compared the model strain genomes with a few sample genomes [24,25,26,27]. However, with the rapid development of high-throughput sequencing technology, hundreds to thousands of different sample genomes can be obtained in a short time. Pangenomics analysis can efficiently capture the distribution of gene families across entire populations and is combined with functional annotation to understand the functional differences of genes in varying group scales. The tools related to bacterial pangenomes are continuously being updated and published [28,29]. The concept of comparative pangenomics was introduced based on the analyses of 12 different pathogen species from multiple perspectives, which systematically introduced the genetic and functional diversity within species [30]. This study primarily focused on *B. subtilis* and 14 other species with economic and research value from the NCBI Refseq database, using comparative pangenomics to parse the genomic diversity and function of different species. It aimed to provide the guidance for functional exploration of the Bs group members, explore the biological drivers behind species openness differences, and highlight the crucial role of genomic instability factors and mobile elements in pangenome openness.

## 2. Materials and Methods

### 2.1. Data Source

All the *B. subtilis* group (Bs group) genome data utilized in this study were downloaded from the NCBI Refseq database, amounting to a total of 2091 genome datasets. The 15 species of the Bs group were identified based on annotations from the NCBI Taxonomy database, whose status was verified according to the List of Prokaryotic names with Standing in Nomenclature (LPSN). Detailed genome data for the species of the Bs group involved in this study are presented in Table 1. The *B. subtilis* dataset comprised a total of 585 genome samples, whereas the genome data for the other 14 species included a total of 1506 genomes.

### 2.2. Genome Data Quality Control and Taxonomic Confirmation

CheckM v1.2.1 [31] was employed in this study to filter the genomes, setting the criteria for genome completeness at above 90% and contamination at less than or equal to 5%. The genomes meeting these criteria were selected for further analysis. Afterwards, Prokka v1.12 [32] was used to annotate the genome data, specifying the domain as Bacteria and the genetic code as 11.

Prior to constructing the pangenome for the species of the Bs group, official tools provided by the Genome Taxonomy Database (GTDB) were used to eliminate genome data samples with clear classification errors. GTDB-Tk v2.3.2 [33] was utilized with default parameters to complete the species classification of all the Bs group genomes. Then, custom scripts were used to extract species information and exclude genome samples with significantly different species annotations. Ultimately, 2072 genome datasets that met the quality standards were obtained from the original 2091 for pangenome construction.

### 2.3. Pangenome Assembly

Panaroo v1.3.4 [34] was used to systematically construct the pangenomes for the 15 species of the Bs group, as well as for the combined genomes. The parameters chosen were: “--clean-mode strict -a core --aligner mafft --core_threshold 0.99 --merge_paralogs” to strictly control the number of core, accessory, and rare genes and minimize the impact of paralogous genes on gene family analysis. The default clustering threshold parameters were set to “--threshold (0.98) and --len_dif_percent (0.98)”.

Through quantification and classification, the definitions of the core genes (present in at least 99% of the species’ pangenome data), accessory genes (distributed between 15 and 99%), and rare genes (less than 15% of the non-redundant genes for the species) were emphasized.

### 2.4. Pangenome Functional Enrichment Analysis

After constructing the pangenomes for different species, custom scripts were used to obtain core, accessory, and rare gene annotations for each species. The R package clusterProfiler v4.4.4 [35] was then employed to perform GO and KEGG enrichment analyses based on a background gene set. The uniform parameter settings were: “pvalueCutoff = 0.05, pAdjustMethod = ‘BH’, qvalueCutoff = 0.05”, where ‘BH’ refers to the Benjamini and Hochberg method for multiple testing correction.

The enrichment results for COG were based on Fisher’s exact test and Bonferroni correction, applied through custom scripts. The value ‘LOG2odds’ reflects the relative magnitude of two ratios, rather than a direct multiple relationship. For example, when comparing core genes to the background set, a positive LOG2odds value indicates enrichment in the core gene set relative to the background, a negative value indicates enrichment in the background set, and a value between 0 and 1 indicates a degree of enrichment but not to the extent of doubling.

The conversion from GO numbers to functional descriptive terms in GO enrichment is as follows: Load the GO description file (see Supplementary Materials) and use R to extract the contents of the GO_IDs column and description column; perform enrichment using the enricher function in the clusterProfiler v4.4.4 [35] package, passing the description file as the TERM2NAME variable.

Regarding significance, this study requires that the q-value remains below the statistical threshold of 0.05 after either the Benjamini and Hochberg method or Bonferroni correction. A result is considered significant only if it meets the former criterion.

### 2.5. Calculation of Pangenome Openness Parameters

The calculation of the openness of the pangenome was based on custom Python v3.7 scripts and Heaps’ Law [36]. Heaps’ Law is formulated as follows:

P = k ∗ n *^λ^*, (1)

where P represents the size of the non-redundant gene set in the pangenome, n represents the number of genomes, k and lambda represent the fitting parameters. The exponent *λ* is indicative of the pangenome’s openness, with a theoretical *λ* > 0 signifying an open pangenome. The utilization of Heaps’ Law, originally identified in linguistics to describe the empirical correlation between the unique words encountered and the total documents reviewed, has found a similar pattern in the realm of bacteria genomics [36]. In our custom script, random sampling based on the number of genomes for each species was performed to calculate the size of the non-redundant gene set at the current sampling size and thus obtain sample points for *λ*. This sampling and calculation process was repeated 50 times, and Heaps’ Law was fitted according to the different numbers of genomes, resulting in the average and standard deviation of *λ* for the species.

### 2.6. Acquisition and Analysis of Genome Stability Factor Data

For the nine categories of genome stability factors, data for the endonucleases, recombinases, repair systems, SOS systems, and toxin–antitoxin-related genes were obtained by keyword searches in the gff files annotated by Prokka v1.12 [32]. Information on prophages and integrases was based on predictions and quality control filtering from genomad [37], with custom scripts used to gather corresponding start sites, lengths, and other data.

Predictions of transfer mobile elements and replication/recombination/repair (RRR) mobile elements, which have been validated by multiple sources, were made using the mobileOG database [38]. Key parameters for mobileOG are set (escore = 1 × 10^−20^, pidentvalue = 90, queryscore = 90) to ensure result accuracy, completeness, and coverage of the results. On this basis, custom scripts are utilized for data processing, information extraction, and visualization.

## 3. Results

### 3.1. Significant Differences in Pangenomes Was Observed among Members of the Bs Group

**Figure 1:**
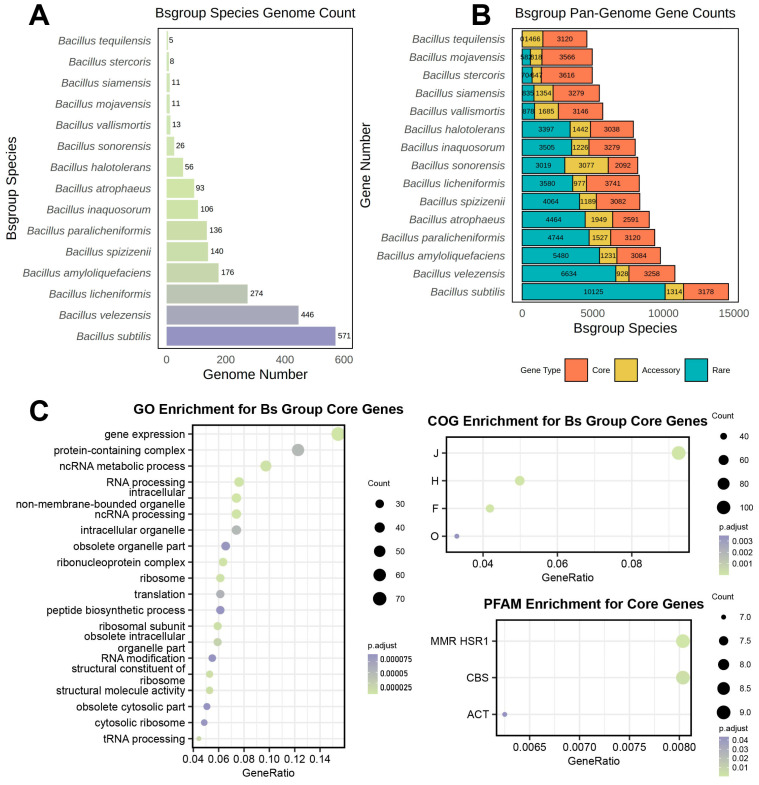
Genome quantity, non-redundant gene set size and core gene functional enrichment results in the Bs Group. (**A**) Number of genomes used for constructing the pangenome of Bs group members; (**B**) size of the non-redundant gene set for the pangenomes corresponding to Bs group members, with orange representing core genes, yellow for accessory genes, and blue for rare genes. (**C**) All terms have *p*-values that pass the hypergeometric distribution test and remain significant after Benjamini and Hochberg correction, indicated as p.adjust in the figure; the x-axis represents the proportion of the corresponding pathway gene quantity, and the bubble size indicates the number of related genes.

In this research, we conducted a precise identification of various species within the Bs group by relying on the valid species names listed in the NCBI Taxonomy database and the authoritative List of Prokaryotic names with Standing in Nomenclature (LPSN). Quality control of these 15 species was uniformly conducted using CheckM v1.2.1, and genomes with over 90% completeness and less than 5% contamination were retained. This process led to the exclusion of two genomes from *B. spizizenii* and two from *B. stercoris*. Then, potential misclassifications of species in the NCBI database were corrected based on the GTDB, resulting in the removal of 14 genome samples from the Bs group and 1 genome sample from *B. vallismortis*. The remaining 2072 genome datasets that met the quality standards were used for further analysis. Based on the 2072 genome datasets after quality control, pangenomes for individual species and for all the combined genome data were constructed using Panaroo v1.3.4. The number of genomes involved in pangenome construction, the scale of the non-redundant gene set of the pangenome, and the number of gene types were analyzed (Figure 1).

Contrary to traditional understanding, the size of the non-redundant gene set of the pangenome did not show a clear linear relationship with the number of genomes (Figure 1A,B). For example, *B. sonorensis*, with only 26 genome samples, had the smallest core gene set and the largest accessory gene set among the 15 species. The non-redundant gene count for this species reached 8188 and was comparable to *B. inaquosorum* (8010) and *B. licheniformis* (8298), which were constructed from 106 and 274 genome samples, respectively, and represented 4.08 and 10.54 times the number of *B. sonorensis*.

To investigate the functional enrichment of the core genes shared among the Bs group species, a pangenome was created using the 2072 genome datasets, resulting in 1141 core genes. These were subjected to enrichment analysis using the Clusters of Orthologous Groups of proteins (COG), Gene Ontology (GO), and the Protein family database (Pfam) to understand the functional purposes of the core genes shared by all the Bs group members. The results are displayed as follows (Figure 1C).

The GO enrichment analysis indicated that the core gene functions of the Bs group were mainly enriched in RNA synthesis and metabolism, essential life activities, and protein synthesis, as well as protein processing and organelle synthesis. Furthermore, the COG enrichment analysis showed that the core genes were mainly enriched in categories J (translation, ribosomal structure, and biogenesis), H (coenzyme transport and metabolism), F (nucleotide transport and metabolism), and O (posttranslational modification, protein turnover, and chaperones), which corresponded with the GO enrichment analysis, indicating that the shared core genes of the Bs group members were significantly enriched in these functions. In summary, the functional enrichments of COG and GO suggested that the highly conserved genes among all the Bs group members were primarily associated with basic life processes; hence, they were highly similar and conserved. Additionally, Pfam enrichment analysis identified significant enrichment in the MMR_HSR1, CBS, and ACT domains within the shared core genome of the Bs group species, suggesting that the shared core gene set emphasized processes such as DNA mismatch repair, energy metabolism, and regulation of amino acid biosynthesis.

### 3.2. COG Enrichment Based on Pangenome Revealed High Conservation of Core Genes in Bs Group

**Figure 2:**
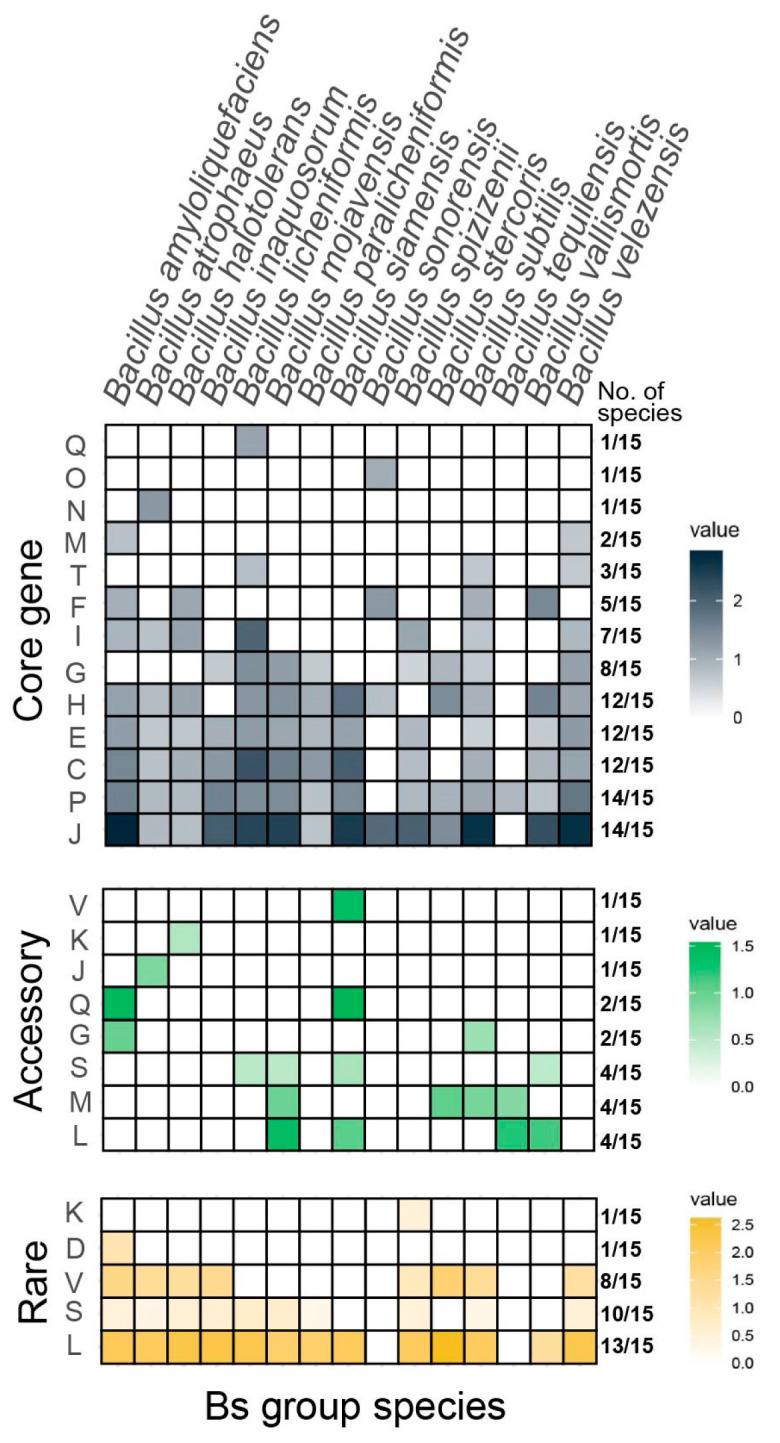
COG enrichment results for different types of gene sets in Bs group members.

COG enrichment analysis of individuals was conducted for the Bs group members, with the results for the three types of genes across the 15 species (Figure 2).

The figure represents the COG functional enrichment of the core genes, accessory genes, and rare genes for different species from top to bottom, with the value represented as LOG2odds. Values greater than 0 imply significant enrichment in these COG functions, with larger values indicating higher levels of enrichment. All the colored blocks have Fisher’s exact test significance with a *p*-value of less than 0.05 and a Bonferroni corrected *p*-value of less than 0.05. The proportion indicated on the right side of the figure represents the number of species in the Bs group with significant enrichment for the corresponding term.

The majority of core genes (14/15) were enriched in key terms, such as J (translation, ribosomal structure, and biogenesis) and P (inorganic ion transport and metabolism). The genes involved in J included the translation initiation factor IF-1 (*infA*), the genes encoding large ribosomal subunit proteins (*rplQ*), the micro-ribonuclease gene *3mrnC*, the arginyl-tRNA synthetase gene (*argS*), and the HTH-type transcriptional repressor gene (*ytrA*), all of which pointed to conservative life processes and functions, which were significantly enriched in the core genes of most of the species in the Bs group. Additionally, it was found that the core genes of *B. licheniformis* were significantly enriched in COG function Q (biosynthesis, transport, and catabolism of secondary metabolites), emphasizing the potential for secondary metabolite exploration in nearly all the genomes of the species; the core genes of *B. sonorensis* were significantly enriched in function O (posttranslational modification, protein turnover, and chaperones); and the core genes of *B. atrophaeus* were significantly enriched in function N (cell motility). The core genes of seven species, including *B. amyloliquefaciens*, *B. licheniformis*, *B. subtilis*, and others, were significantly enriched in category I (lipid transport and metabolism), suggesting a potential for the exploration of widespread lipopeptide components within these species.

For the accessory genes, five species, including *B. inaquosorum*, *B. paralicheniformis*, *B. sonorensis*, *B. spizizenii*, and *B. velezensis*, showed no significant COG term enrichment. It was also noted that *B. siamensis* had accessory genes significantly enriched in COG function V (defense mechanisms), function Q (biosynthesis, transport, and catabolism of secondary metabolites), and function M (cell wall/membrane/envelope biogenesis), which were less enriched in core genes. Additionally, the accessory genes of *B. amyloliquefaciens* were enriched in categories Q (secondary metabolites biosynthesis, transport, and metabolism) and G (carbohydrate transport and metabolism), indicating that a significant proportion of strains, although not in most of the samples, exhibited notable capacities for secondary metabolite production as well as carbohydrate transport and metabolism. The accessory genes of *B. mojavensis*, *B. stercoris*, *B. subtilis*, and *B. tequilensis* were enriched in category M (cell wall/membrane/envelope biogenesis), whereas this enrichment in category M was not observed in the core gene enrichment of the corresponding species.

The COG enrichment analysis of the rare genes revealed that a significant proportion of species (13/15, 86.67%) were consistently enriched in function L (replication, recombination, and repair) and 53.33% (8/15) in function V (defense mechanisms). It was further discovered that the rare genes of *B. spizizenii* were significantly enriched in function K (transcription), while the rare genes of *B. amyloliquefaciens* were significantly enriched in function D (cell cycle control, cell division, and chromosome partitioning).

### 3.3. GO Enrichment Based on Pangenome Revealed Significant Differences of Accessory Genes and Species-Specific Core Genes in the Bs Group

**Figure 3:**
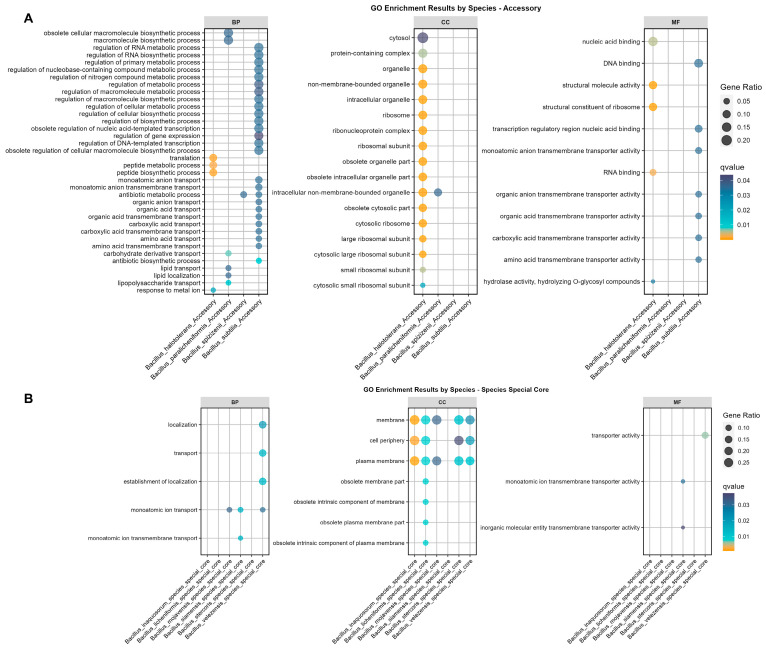
GO enrichment results for Bs Group species-specific core gene and accessory gene set. (**A**) GO enrichment results for accessory gene sets specific to members of the Bs group, from left to right, are for the GO enrichments in biological process (BP), cellular component (CC), and molecular function (MF) categories, with four species within the Bs group showing significant GO enrichment results. (**B**) Enrichment results for species-specific core gene sets, from left to right, are for the GO enrichments in biological process (BP), cellular component (CC), and molecular function (MF) categories, with six species showing significant GO enrichment results. The q-value is based on the hypergeometric test and adjusted using the Benjamini and Hochberg method, with only significant results displayed in the figure. The orange sections indicate a q-value of less than 0.01. The descriptive terms for GO enrichment are converted using the enricher function, as detailed in Section 2.4.

Considering the broad scope of the COG functional annotation terms, the enrichment analysis of GO was combined with COG to provide a comprehensive analysis of functional enrichment for individual species. All the non-redundant gene sets were divided into four categories: core genes for individual species, accessory genes, rare genes, and a species-specific core gene set (excluding the 1141 core genes shared among the 15 species) (Figure 3, Supplementary Figures S1 and S2). The GO enrichment analysis performed against the background set of individual species revealed the following (Figure 3).

The accessory gene set enrichment for the Bs group members showed that only four species exhibited significant enrichment (Figure 3A). The accessory genes of *B. halotolerans* were notably enriched in translation, peptide biosynthesis and transport processes, response to metal ions, ribosomal structure, and RNA binding, matching the COG enrichment in function K (transcription) (Figure 2). Also, the accessory genes of *B. paralicheniformis* were enriched in the polymer biosynthesis, carbohydrate derivative transport, lipid transport, and lipopolysaccharide transport pathways. Furthermore, the accessory genes of *B. spizizenii* were enriched in antibiotic metabolic pathways. Lastly, the enrichment of accessory genes showed a significant diversity within the species of the Bs group that was mainly associated with biosynthetic processes, including RNA metabolic process regulation, nitrogen compound metabolic process regulation, macromolecule metabolic process regulation, cellular biosynthesis, organic anion transport, organic acid and carboxylic acid transport, and antibiotic biosynthetic process pathways.

The species-specific enrichment of core genes indicated significant GO term enrichment in six species (Figure 3B). Among them, *B. siamensis*, *B. velezensis*, and *B. mojavensis* exhibited significant enrichment in the monoatomic ion transport pathways. Additionally, *B. siamensis* and *B. velezensis* also demonstrated enrichment in the molecular function (MF) categories associated with transporter pathways. Considering that core genes account for 99% or more of the species’ genome samples, we speculated that the significant enrichment of transport protein-related pathways played an important role in the adaptability and evolutionary survival of the aforementioned species. Furthermore, the enrichment from the cellular component (CC) showed that species-specific core genes were mainly enriched in the cell membrane (5/6), periphery (4/6), and plasma membrane (5/6), matching the key terms of transport and conveyance in the BP and MF functions (Figure 3B).

### 3.4. KEGG Enrichment Based on Pangenome Revealed Medicinal Potential in the Bs Group

**Figure 4:**
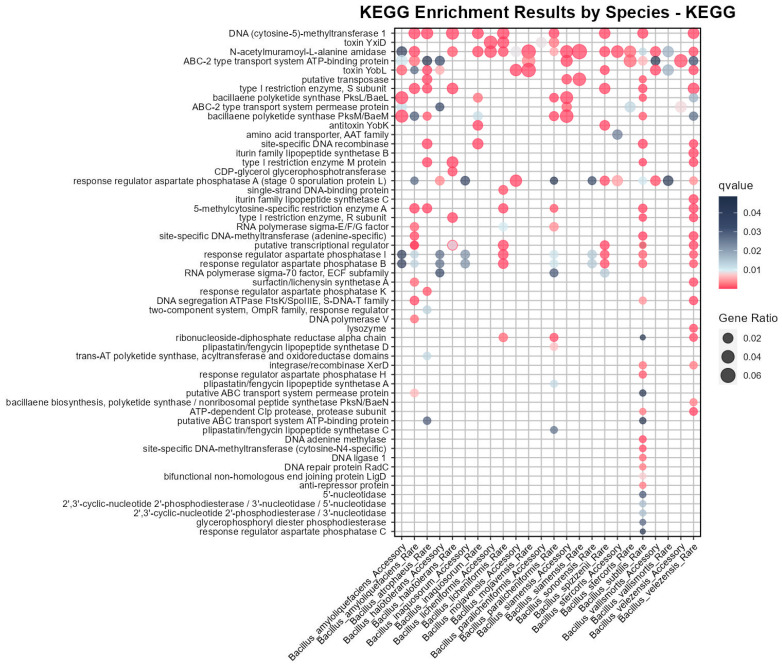
Pangenome KEGG enrichment analysis of the Bs group members.

While COG and GO enrichment analysis focused on standardized interpretations and functional classifications of individual genes, they did not adequately characterize the specific metabolic networks or pathways that the genes participated in. To solve this, KEGG enrichment analysis, which is similar to that of COG and GO, was performed for the Bs group members. The results indicated that only the enrichment of the core genes, accessory genes, and rare genes from *B. tequilensis* lacked significance, while the majority from the other species (14/15) showed KEGG pathway enrichment for the accessory or rare genes (Figure 4).

KEGG enrichment for the rare genes of Bs included DNA adenine methylase and site-specific DNA methyltransferase, which corresponded to the DNA methylation pathway enrichment (Supplementary Figure S2), and in KEGG enrichment, the rare genes of Bs were also enriched in aspartate phosphatase H and ATP-dependent Clp protease. The rare genes of *B. velezensis* were specifically enriched in Iturin-like lipopeptide-related pathways, such as Iturin lipopeptide synthase B and synthase C, which had significant antifungal activity and were used in biopesticides and plant growth regulators [39]. Additionally, *B. velezensis* was specifically enriched in bacillaene biosynthesis and lysozyme synthesis and, along with *B. amyloliquefaciens*, was enriched in Surfactin/Lichenysin synthetase A. Surfactin possessed potent antimicrobial capabilities and was widely used in the fields of biopesticides and pharmaceuticals [40]. These results had not been reflected in previous COG and GO enrichments, emphasizing the important application value of some strains of *B. velezensis*. Rare genes of *B. paralicheniformis* were specifically enriched in Plipastatin/Fengycin lipopeptide synthase A, C, D, which were similar to the Iturin family and primarily used for inhibiting the growth of yeasts and *Staphylococcus aureus* [41].

The x-axis represents the gene sets corresponding to species with significant KEGG enrichment results, with 14 species in total and only *B. tequilensis* lacking enrichment results. The y-axis represents functional description terms, the bubble size indicates the proportion of the pathway result, and the q-value is based on the hypergeometric distribution test and adjusted using the Benjamini and Hochberg method, with significant results displayed in pink, indicating a q-value of less than 0.01. All bubbles appearing in Figure 4 meet the corrected significance threshold requirements. Note that there are two bubbles associated with the description ‘putative transcriptional regulator’ in the context of the ‘*B. halotolerans* rare genes’. This is not an error, as it involves simultaneous enrichment in pathways: ko:K07727 and ko:K07729.

In addition to the species-specific KEGG enrichment mentioned above, it is important to note that the subsequent denominator of 24 represents a total of 24 columns of significant results for different species and gene types in Figure 4, to avoid confusion with the 15 species of the Bs group. The KEGG enrichment results shared by a larger number of species and gene types (with the same functional description term corresponding to a count on the x-axis of Figure 4 that is greater than or equal to 10) were as follows:

Regulatory agent aspartate phosphatase A was enriched in a large number of species (12/24), and aspartate phosphatase B was enriched in I (10/24), which was consistent with the existing research conclusions: aspartate-representative protein phosphatases played an important role in the spore germination, environmental response, and metabolic regulation networks of *Bacillus* [42]. In addition, a significant enrichment of N-acetylmuramoyl-l-alanine amidase (18/24) was observed in the Bs group, playing a crucial role in bacterial growth as well as in the synthesis and breakdown of the cell wall, which was potentially related to the biofilm formation in the *Bacillus* species. 

### 3.5. Core Genes and Rare Genes Jointly Contributed to the Pangenome Openness among Bs Group

Research reports have suggested that Heaps’ law could be applied to pangenomics studies to quantify and compare the pangenome openness of different species [30]. Therefore, this study constructed a script based on Heaps’ law and systematically quantified the pangenome openness of each member in the Bs group, with the subsequent results referring to the pangenome openness assessment parameter using “*λ*”.

**Figure 5:**
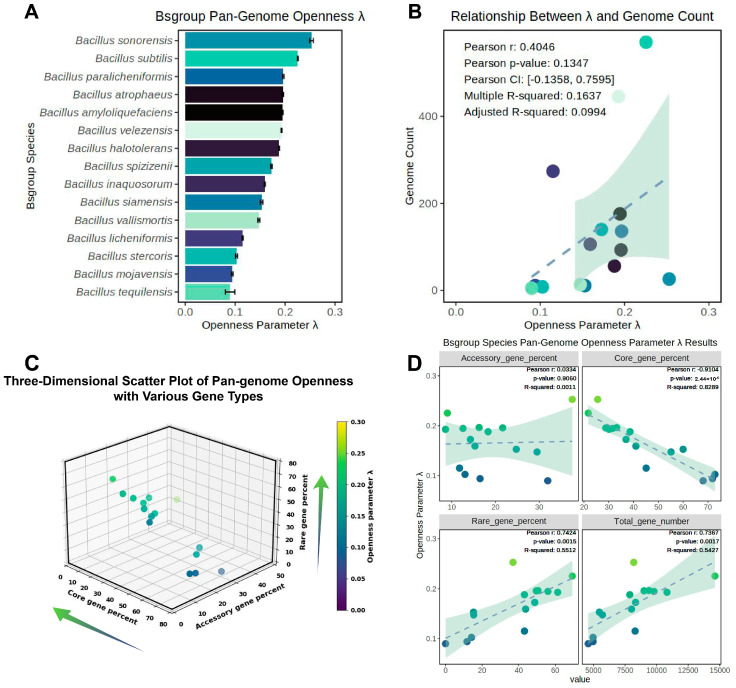
Exploration of the relationship between pangenome openness and genome quantity in the Bs group. (**A**) The magnitude and variance of the pangenome openness index *λ* for the Bs group; (**B**) study of the correlation between *λ* and the number of genomes used in pangenome construction. Pearson’s correlation coefficient and linear model were used to explore the relationship between variables, with related parameters annotated in the upper left corner of Figure 5B. (**C**) Scatter distribution of the percentage of core, accessory, and rare genes with the pangenome openness index *λ*; (**D**) the linear correlation between the percentage of core, accessory, and rare genes, the quantity of the complete non-redundant gene set for individual species, and *λ*, with Pearson’s correlation coefficient, *p*-value, and R-squared of the linear model annotated in the upper right corner. The colors in Figure 5A,B were matched, with different colors used to distinguish different species; Figure 5C,D shared matching colors, with the color scheme in Figure 5D following the same pattern as in Figure 5C, where lighter colors represented greater species openness index; the direction of arrows in Figure 5C indicated the direction of increasing openness along the corresponding axes.

This study calculated the mean and variance of the normalized pangenome openness parameter *λ* for each species in the Bs group and explored the relationship between *λ* and the number of genomes involved in pangenome construction. The results revealed that all the members of the Bs group conceptually belonged to open pangenomes (*λ* > 0), with significant differences in *λ* between different species, ranging from 0.090 to 0.253 (Figure 5A). Ideally, all the species of the Bs group would belong to open pangenomes, but the openness differences between species were clearly significant. To quantitatively explore the correlation between the number of genomes involved in pangenome construction and the openness index *λ*, Pearson’s coefficient and the explanatory capacity of the R-squared of the linear model were used for testing (Figure 5B). Although there was a certain correlation between the number of genomes involved in pangenome construction and the openness *λ*, it was not significant (Pearson correlation coefficient of 0.405, *p*-value of 0.135); the R-squared of the linear model was only 0.164 (adjusted R-squared only 0.099), indicating a poor fit with the linear model. Therefore, this study concluded that although the number of genomes might be one factor affecting pangenome openness, it cannot be considered a strong or significant factor. For example, *B. sonorensis* had only 26 genomes but possessed the most open pangenome among the 15 species; *B. licheniformis* had the third-highest number of genomes among the member species (274), but its openness index was only 0.115, ranking fourth from the bottom among the 15 species.

The relationship between the openness *λ* of the pangenome within the same species and the percentage of core, accessory, and rare gene sets, as well as the quantity of the non-redundant gene set in the pangenome, was explored (Figure 5C,D).

The three-dimensional scatter plot revealed that the change in *λ* index was most evident along the core gene percent axis, with a lower core gene percent corresponding to a higher openness index *λ* (Figure 5C). There was also a gradient decline along the rare gene percent axis, with a higher rare gene percent correlating with a higher openness index *λ*. However, no directional gradient change was observed along the accessory gene percent axis. Based on this analysis, a correlation was found between the *λ* index and the percentage of the three gene categories, as well as the quantity of non-redundant genes in the pangenome of individual species (Figure 5D). The results were consistent with the preliminary observations from Figure 5A: there was a significant correlation between the core genes and rare genes with the *λ* parameter, with a highly significant strong negative correlation between the core genes and *λ* (*p*-value < 2.44 × 10^−6^ and a correlation coefficient of −0.910, and the explanatory power of the linear model R-squared reached 0.829). There was a significant positive correlation between the rare genes and *λ*, with a correlation coefficient of 0.742, and the R-squared of the linear model reached 0.551. There was no significant correlation between the accessory gene percent and *λ*, and a significant positive correlation was found between the quantity of the complete non-redundant gene set in the pangenome of individual species and *λ*. Therefore, this study demonstrated that the lower the percentage of core genes and the higher the percentage of rare genes, the more open the pangenome; the more non-redundant genes in the overall pangenome, the more open it was.

### 3.6. The Distribution of Integrase Genes Was Significantly Correlated with the Openness of the Bs Group

The percentage of core and rare genes were key indicators affecting the openness parameter *λ* of the species, but this was insufficient to clearly articulate the biological drivers behind the differences in openness between species. This study posited that the differences in pangenome openness were fundamentally related to genome stability, and many factors influencing bacterial genome stability have been systematically reported [43]. Building on this, the current study selected nine systems for exploration, including genes related to endonucleases, recombinases, repair systems, SOS systems, toxin–antitoxin systems, prophages, integrases, transfer mobile elements predicted by the mobileOG database, and replication/recombination/repair mobile elements, to investigate the driving forces behind the openness parameter *λ* of the Bs group members’ pangenomes.

**Figure 6:**
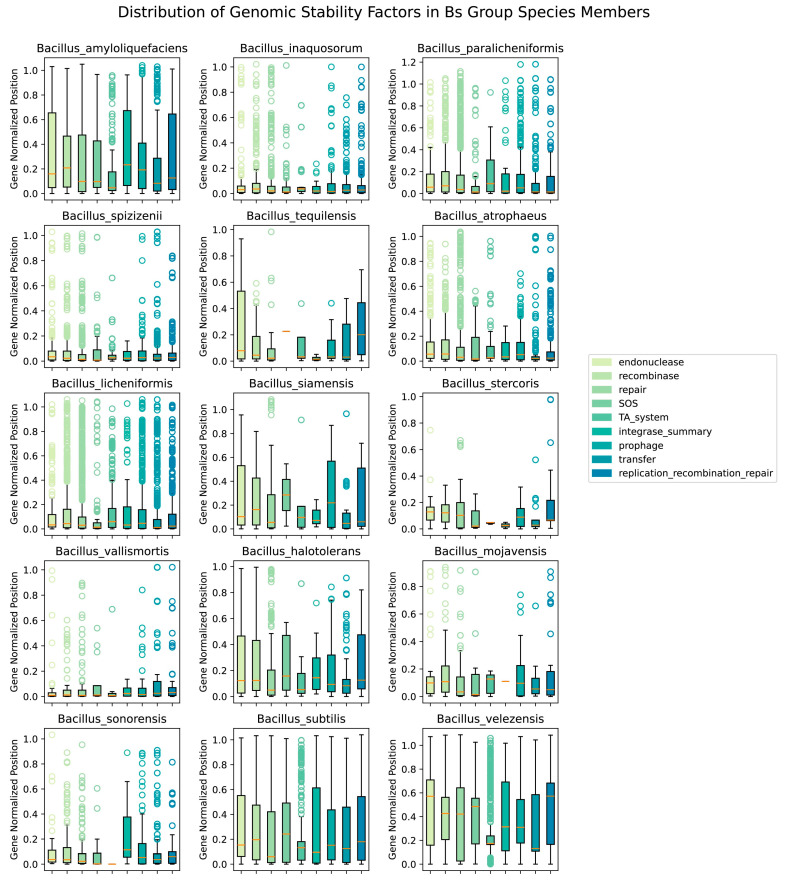
Boxplot of initiation site distribution of 9 Categories influencing openness factors in the Bs group.

To explore the correlation between the openness parameter *λ* and the aforementioned nine factors, the study was conducted to examine the number of genes related to these factors in different species and their distribution of starting sites. The result displayed the corrected distribution positions of the genes from the nine factors across the genomes of different species in the Bs group (Figure 6).

The figure includes 15 Bs group species members, each corresponding to the boxplot of nine categories of genes influencing genome stability on the right. The y-axis represents the relative distribution of the initiation sites of the related genes compared to the average genome size of the species, which is typically in the range of 0–1; however, some points exceed 1, as a few sample points have absolute initiation sites on the genome that are larger than the average genome size of the species. The orange lines in Figure 6 represented the mean values of the distribution data for corresponding factors.

There was a clear difference in the average genome size among different species of the Bs group: the largest, *B. sonorensis*, had an average genome size of 4.6 Mb, and the smallest, *B. tequilensis*, had an average genome size of 3.9 Mb. Contrary to initial expectations, the overall correlation between the corrected initiation sites of the different influencing factor genes and the openness of the species’ pangenomes was not clear. For example, the two species with the greatest openness in the Bs group, Bs and *B. sonorensis*, had a large difference in the number of genomes contributing to their pangenomes (a difference of 545 genome datasets), resulting in *B. sonorensis* having influencing factor distributions closer to 0 (median ≤ 0.10, boxplot limits near 0.20), while Bs had a broader distribution (median ≤ 0.20, boxplot limits near 0.40). To minimize the impact of the number of species genomes, we focused on comparing three more open species, *B. siamensis* (11), *B. sonorensis* (26), and *B. vallismortis* (13), and three more closed species, *B. stercoris* (8), *B. mojavensis* (11), and *B. tequilensis* (5), with the number in parentheses indicating the number of genomes. It could be seen that the distribution of factors such as prophage and integrase followed a pattern: the greater the openness of the species’ pangenome, the more evenly distributed the related factors were on the genome (Figure 6).

**Figure 7:**
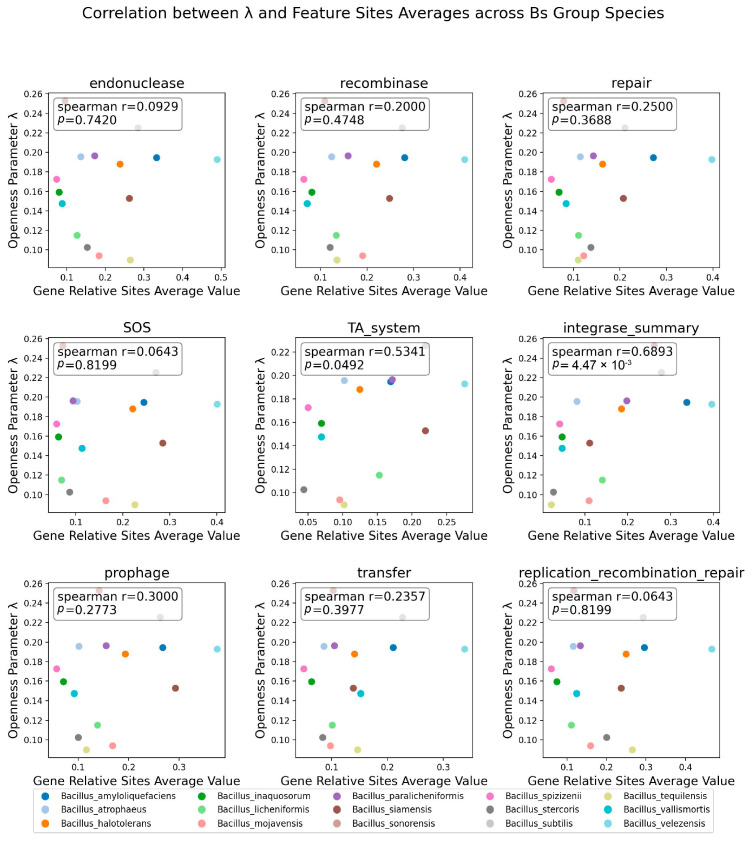
Spearman correlation between positions of 9 influencing openness factors and *λ*. The influencing factors are labeled at the top of the subfigure; the x-axis represents the average relative position of the distribution correction of the related factors on the genome, and the y-axis represents the openness index *λ*. The Spearman correlation coefficient and *p*-value between points are displayed in the upper left corner of the image.

To further quantify the existence of the above pattern, the study calculated the average relative positions of the nine factors for each species and used the Spearman correlation coefficient to assess the relationship between the averages and the *λ* parameter (Figure 7). Among the corrected distribution positions of the genes related to the nine factors affecting genome stability, the distribution positions of the TA system and integrase genes on the genome were found to have a significant correlation with the openness *λ* of the Bs group species members, with an integrase Spearman correlation coefficient of 0.689 and a *p*-value of 4.47 × 10^−3^. This conclusion also confirmed the phenomenon (Figure 6): the greater the openness of the species’ pangenome, the more uniform the distribution of integrases on the genome.

### 3.7. The Quantity of Prophages and Other Factors Was Significantly Correlated with Openness

**Figure 8:**
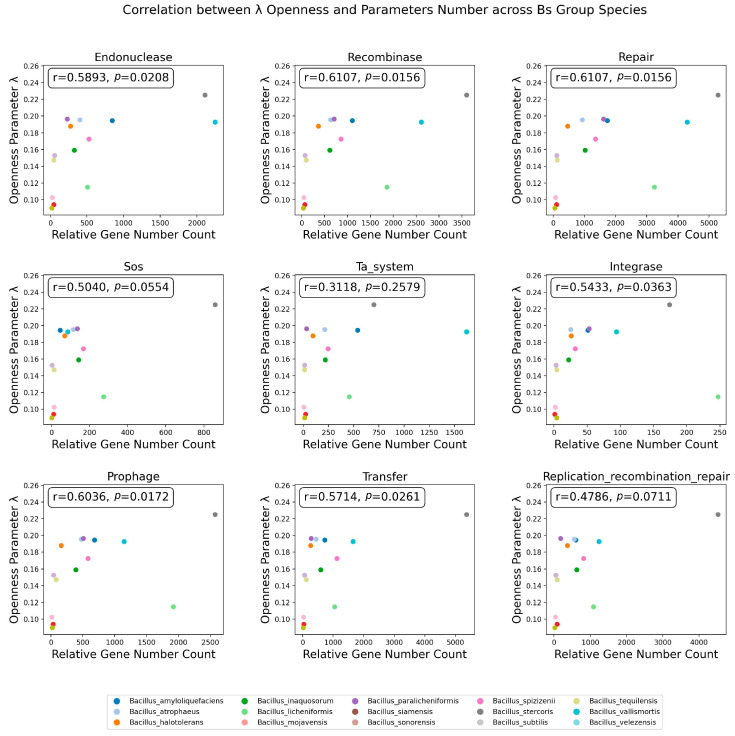
Spearman correlation between the number of genes across 9 Categories and *λ*. The influencing factors are labeled at the top of the subfigure; the x-axis represents the number of related factors in the corresponding species’ genome samples, and the y-axis represents the openness index *λ*. The Spearman correlation coefficient and *p*-value between points are displayed in the upper left corner of the image.

In addition to gene location information, this study also examined the correlation between the number of genes from the nine influencing factors and the openness parameter *λ* of the species’ pangenomes (Figure 8). It was found that 66.67% (6/9) of the assessed genome stability influencing factors had a significant spearman correlation with the *λ* parameter of the Bs group species, while the number of genes from SOS emergency damage repair, the TA system, and the replication/recombination/repair-related mobile elements did not have a significant correlation with the *λ* of the Bs group species. The average spearman correlation coefficient for the six factors significantly correlated with *λ* and had a value of 0.588, indicating that among the nine factors affecting genome stability explored in this study, the number of genes related to endonucleases, recombinases, repair systems, prophages, integrases, and transfer mobile elements was the main driver of the openness of the species’ pangenomes.

## 4. Discussion

The 15 species comprising the Bs group played significant roles in the current fundamental scientific research, synthetic biology modification, and industrial application. However, systematic functional comparisons between these species, especially in the field of comparative pangenomics, were relatively scarce. The concept of comparative pangenomics, which involved the comparative analysis of the pangenomes from various populations of organisms, such as pathogens, increasingly gained prominence. This study aimed to fill this gap by systematically investigating the functional differences between pangenomes of the different Bs group species, the extent of pangenome openness, and the underlying driving forces.

### 4.1. Comparative Pangenomes and Enrichment Analysis Facilitated Resource Mining in the Bs Group

By inputting 2072 genomes from the Bs group members to construct a pangenome, 1141 core genes were identified. The enrichment results of these core genes suggested that members of the Bs group adopted a relatively conservative strategy in maintaining genome integrity, optimizing metabolic efficiency and regulating nutrient utilization. This might also contribute to the wide ecological distribution of species such as Bs and *B. velezensis*.

Effective ion transport systems that helped bacteria maintain normal metabolic activity and growth under conditions such as drought, salt stress, and nutrient depletion were identified [44,45,46]. In some species such as *B. velezensis*, *B. mojavensis*, and *B. siamensis*, species-specific core genes were significantly enriched in pathways related to transport proteins and ion transport proteins. Hence, it was hypothesized that the significant enrichment of species-specific core genes in ion transport might be closely related to the unique survival strategies and adaptability of these species, playing a pivotal role in the population formation and evolution of species such as *B. mojavensis*.

Recent studies have shown that a strain of *B. paralicheniformis*, SN-6, could enhance the host’s amino acid metabolism (mainly tryptophan metabolism) and lipid metabolism pathways and serve as a probiotic additive for animals [47]. This result corresponded with the GO enrichment analysis of *B. paralicheniformis*. Considering that accessory genes occupied at least 15% of the population in the species groups, our enrichment analysis highlighted that numerous strains of *B. paralicheniformis* possessed probiotic potential. The accessory genes of *B. spizizenii* were significantly enriched in antibiotic metabolism pathways; this was consistent with the report that members of *B. spizizenii* secrete lanthipeptide antimicrobial substances [48]. Therefore, this study suggested that the antibiotic-related gene pathways of *B. spizizenii* warranted further exploration.

### 4.2. Genome Stability Factors and Mobile Elements Influence the Openness of the Species Pangenomes

This study quantified the openness of the pangenomes of the different Bs group species based on Heaps’ law and investigated the relationship between the distribution, the quantity of genes related to the nine factors proven to affect microbial genome stability, and the openness index [36,43]. Comparing the openness index with the published data, we found that the openness of the Bs group species members significantly differed from the *Lactobacillaceae* family and pathogens such as *E. cloacae*, *S. enterica*, *A. baumannii*, *P. aeruginosa*, *K. pneumoniae*, and *E. coli*. These pathogens’ average *λ* ranged from 0.42 to 0.47; *Lactobacillaceae*’s *λ*, except for *Lactobacillus acidophilus*, was generally between 0.15 and 0.35, while the Bs group members involved in this study ranged from 0.09 to 0.25 [30,49]. Considering the actual meaning of the *λ* parameter, we believed that this phenomenon was due to the Bs group members occupying significantly different ecological niches compared to the pathogens and *Lactobacillus*. The Bs group species had a significantly higher proportion of core genes to maintain a survival advantage in a variety of complex environments; pathogens and *Lactobacillus*, on the other hand, did not require a high proportion of stable core genes; thus, the acquisition and loss of new genes occurred more frequently.

Looking at the biological drivers influencing the openness of the species’ pangenomes, the quantity of transfer, prophages, and other mobile elements in the Bs group members was significantly correlated with the openness coefficient. This implied that mobile elements played an important role in the process of different species acquiring new genes and significantly affected the openness of the pangenome. Given that integrases and other mobile elements were closely associated with horizontal gene transfer between bacteria [50,51], this study provided evidence and reference for the impact of horizontal gene transfer events on the openness of species’ pangenomes. In addition to mobile elements, intrinsic genome instability factors in bacteria were also significantly correlated with the openness of the Bs group species’ pangenomes. We speculated that these factors might be the main driving forces behind the diversity within the Bs group members and their strong ecological adaptability. However, it was clear that pangenome openness was significantly correlated with a variety of different factors, which undoubtedly increased the difficulty of systematically analyzing the driving forces behind species openness. Research on driving forces could be further deepened by adopting causal inference and constructing multifactorial regression models. This study provided some ideas and basic information for understanding and predicting species openness.

## 5. Conclusions

This study, which focused on 15 species within the Bs group, identified significant functional differences among various types of genes across these species through comparative pangenome analysis and functional enrichment. Also, it provided guidance for targeted exploration of certain species, such as the potential for mining secondary metabolites in *B. licheniformis* genome members, the probiotic potential of some strains in *B. paralicheniformis*, etc. The quantification of the openness of the pangenomes among species in the Bs group highlighted that all members possessed open pangenomes, with notable variability in openness among different species. Combined correlation analysis indicates that the number of genes related to endonucleases, recombinases, and repair systems and the distribution of the TA system and integrase-related genes on the genome, as well as the quantity of prophages, integrases, and transferable mobile elements, are the primary biological factors influencing the species’ pangenome openness size.

## Figures and Tables

**Table 1:** Statistics of Bs group species and the number of genomes after quality control.

Species	Formal Genome Number	CheckM Number	GTDB Number	LPSN Message
*B. amyloliquefaciens*	176	176	176	TRUE
*B. halotolerans*	56	56	56	TRUE
*B. mojavensis*	11	11	11	TRUE
*B. siamensis*	11	11	11	TRUE
*B. tequilensis*	5	5	5	TRUE
*B. velezensis*	446	446	446	TRUE
*B. atrophaeus*	93	93	93	TRUE
*B. licheniformis*	274	274	274	TRUE
*B. paralicheniformis*	136	136	136	TRUE
*B. sonorensis*	26	26	26	TRUE
*B. vallismortis*	14	14	13	TRUE
*B. spizizenii*	142	140	140	TRUE
*B. stercoris*	10	8	8	TRUE
*B. inaquosorum*	106	106	106	TRUE
*B. subtilius*	585	585	571	TRUE

Note: Table 1 presents the number of genomes meeting set standards. For *B. subtilis*, 585 genomes remained after the CheckM quality control. Following GTDB taxonomy curation, 571 genomes were retained. LPSN confirms the species’ valid taxonomic status. See Supplementary Table S1 for details.

## Data Availability

Data are contained within the article and Supplementary Materials.

## Supplementary Materials

The following supporting information can be downloaded at: https://www.mdpi.com/article/10.3390/microorganisms12050986/s1, Figure S1: GO enrichment results for Bs group species core gene set, Figure S2: GO enrichment results for Bs group species rare gene set, Table S1: Set sample IDs and species information for 2091 Bs group genome samples, Table S2: Pangenome openness and average genome size of the Bs group species, Table S3: Statistical count of stability factors for Figure 8, Table S4: Data on the mean values of corrected distribution positions for Figure 7.
