# Supplementary material for: Comparative Genomics Unveils Functional Diversity, Pangenome Openness, and Underlying Biological Drivers among Bacillus subtilis Group
Source: Microorganisms. 2024 May 14;12(5):986. doi: 10.3390/microorganisms12050986 (PMC11124052; doi:10.3390/microorganisms12050986)
Supplement: Supplementary file 1 [file microorganisms-12-00986-s001.zip › microorganisms-2985640-supplementary/supp_figureS1&S2.pdf]

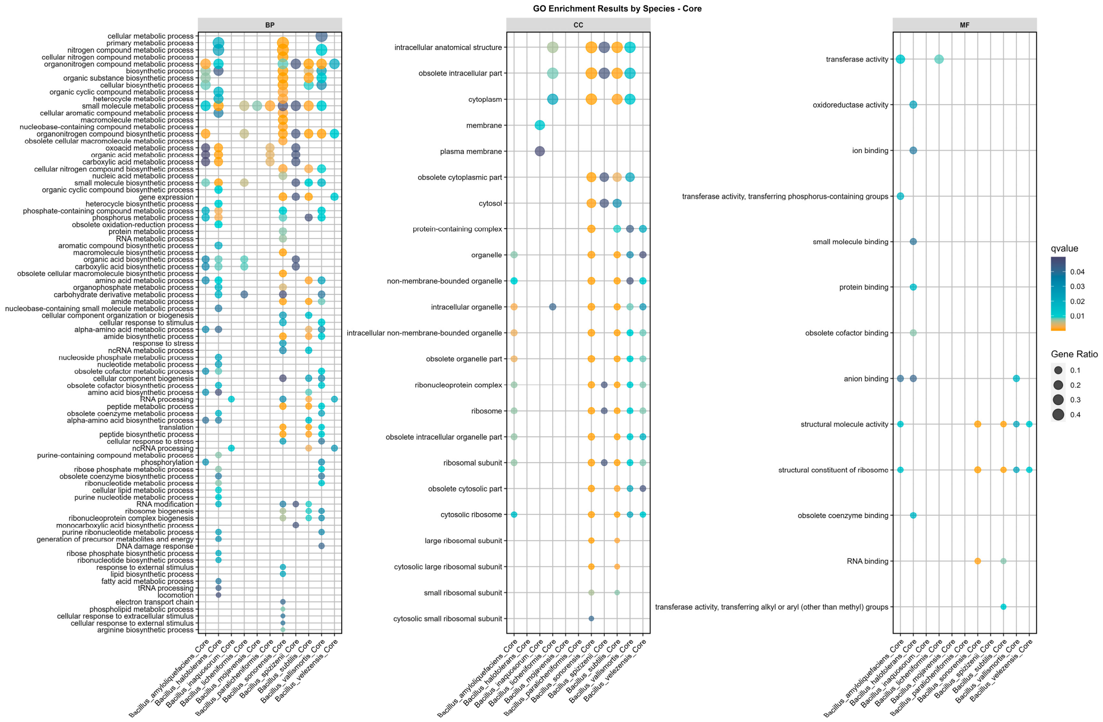

Supplementary Figure S1. GO Enrichment Results for Bs Group Species Core Gene Set

From left to right are the BP, CC, and MF functions of GO enrichment for the Bs group species core gene set, showing only the 11 species with significant GO enrichment results for core genes. The q-value is based on the hypergeometric distribution test and adjusted using the Benjamini & Hochberg method, with significant results displayed in orange, indicating a q-value less than 0.01.

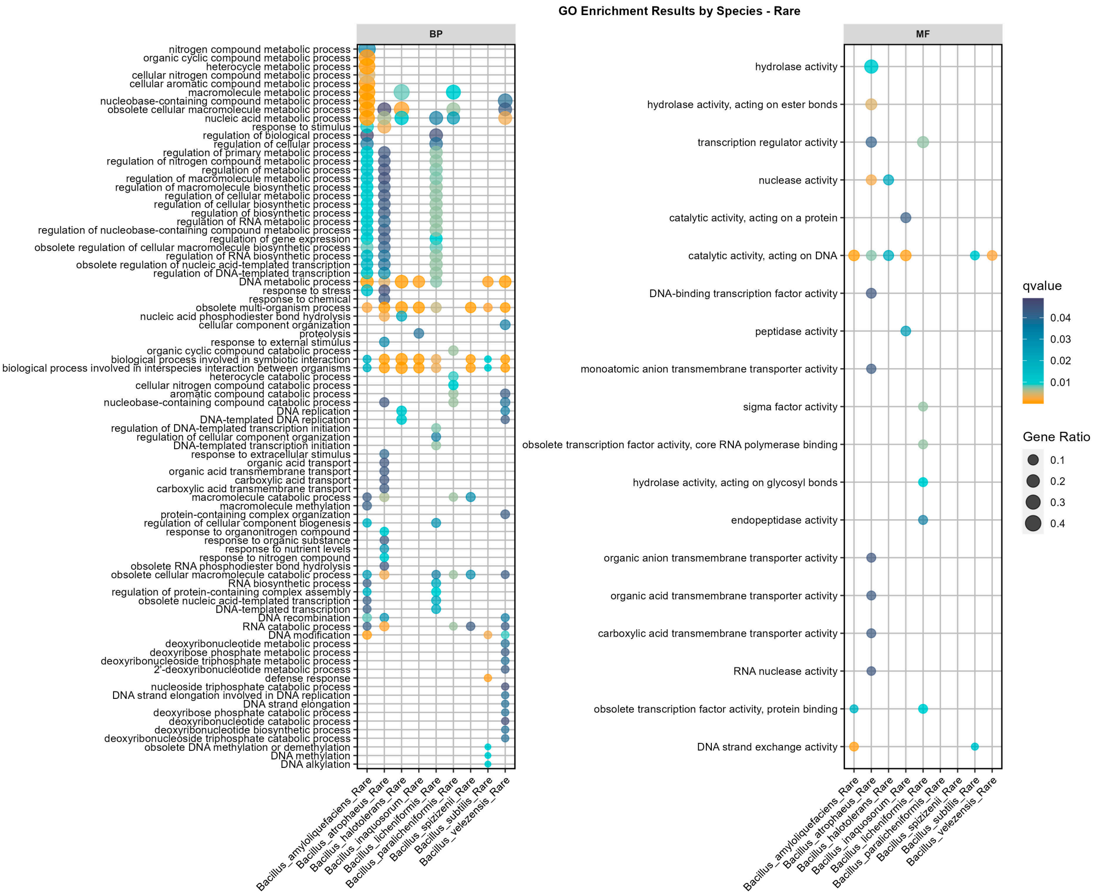

Supplementary Figure S2. GO Enrichment Results for Bs Group Species Rare Gene Set

From left to right, the figure shows the GO enrichment of rare genes in Biological Process (BP) and Molecular Function (MF) categories, with nine species within the Bs group exhibiting enrichment results. The q value is based on the hypergeometric test and adjusted using the Benjamini & Hochberg method, with only significant results displayed in the figure. The orange sections indicate a q value of less than 0.01.
